# Ipilimumab treatment decreases monocytic MDSCs and increases CD8 effector memory T cells in long-term survivors with advanced melanoma

**DOI:** 10.18632/oncotarget.15368

**Published:** 2017-02-16

**Authors:** Yago Pico de Coaña, Maria Wolodarski, Isabel Poschke, Yuya Yoshimoto, Yuan Yang, Maria Nyström, Ulrika Edbäck, Suzanne Eghyazi Brage, Andreas Lundqvist, Giuseppe V. Masucci, Johan Hansson, Rolf Kiessling

**Affiliations:** ^1^ Department of Oncology and Pathology, Cancer Center Karolinska, Karolinska Institutet, Stockholm, Sweden; ^2^ Karolinska University Hospital Solna, Stockholm, Sweden; ^3^ Division of Molecular Oncology of Gastrointestinal Tumors, German Cancer Research Center, Heidelberg, Germany; ^4^ Department of Radiation Oncology, Gunma University Graduate School of Medicine, Maebashi, Gunma, Japan; ^5^ Cancer Immunology and Immunotherapy Center, The Affiliated Hospital of Guiyang Medical College, Guiyang, People's Republic of China; ^6^ Cell Therapies Institute, Nova Southeastern University, Fort Lauderdale, FL, USA

**Keywords:** melanoma, checkpoint blockade, CD8 effector memory T cells, ipilimumab, MDSC

## Abstract

Ipilimumab has revolutionized malignant melanoma therapy, but a better understanding of the mechanisms behind treatment response and adverse effects is needed. In this work, the immune system of ipilimumab treated patients was monitored to investigate potential mechanisms of action that may correlate with treatment outcome. Blood samples from 43 advanced melanoma patients were taken before, during and at the end of treatment. Hematological parameters were measured and flow cytometry analysis was performed in fresh samples within two hours of sample collection. Strong differences in markers CD45RA, CCR7, HLA-DR and CD15 between fresh and cryopreserved samples were observed. Ipilimumab treatment increased absolute lymphocyte counts, eosinophils, effector T cells and their activation status, whilst diminishing the suppressive side of the immune response, acting on regulatory T cells and myeloid derived suppressor cells (MDSCs). These effects were visible after one ipilimumab infusion and, regarding eosinophil counts, correlated with onset of adverse events. Monocytic MDSCs were decreased in response to treatment only in patients with clinical benefit; additionally, patients with a lower frequency of these cells after the first ipilimumab infusion experienced increased overall survival. CD8 effector memory T cell frequencies at the end of treatment were higher in patients with clinical benefit and positively correlated with survival. These data show that a clinical response to ipilimumab not only requires reshaping T cell populations, but additionally involves a reduction in suppressive cells such as monocytic MDSCs. Our work could provide insight on predicting treatment outcome, assisting clinicians in offering the best personalized therapeutic approach.

## INTRODUCTION

The role of the immune system in the surveillance and elimination of several types of human cancers has been known for a long time, but not until recently has this knowledge been transformed into clinically useful cancer therapies. The recent surge in cancer immunotherapies is based on a better knowledge of the regulation of the interaction between cancer cells and the immune system via immunologic checkpoints. Antibody blockade of the checkpoints cytotoxic T lymphocyte-associated antigen 4 (CTLA-4), and the programmed cell death protein 1 pathway (PD1/PD-L1) have demonstrated efficacy in a number of malignancies. Specifically, blocking the checkpoint receptor CTLA-4 with the monoclonal antibody ipilimumab is the first treatment that has been proven to improve overall survival in patients with advanced melanoma. After initial reports that this treatment produced responses in 13% of patients [1], phase III clinical trials demonstrated that ipilimumab reduced the risk of death by 32% [2]. Moreover, ipilimumab has been shown to increase long term survival in over 20% of patients with advanced melanoma [3], although adverse events, while manageable, are frequent [4]. Currently it is of major interest to establish well-documented predictive biomarkers that may allow for pre-screening of patients as well as pharmacodynamic markers assisting in patient monitoring and adverse event management. Given the emerging combination therapy strategies combining ipilimumab with other agents such as PD-1/PD1-L checkpoint blockade [5], such biomarkers would be of great usefulness when ipilimumab is used as a single or combined therapeutic agent in the treatment of advanced melanoma.

Although the detailed mechanisms of action of CTLA-4 blockade with ipilimumab are not fully understood, they can be divided into two main types: Cell intrinsic mechanisms acting *in cis*, and cell extrinsic mechanisms acting *in trans*. *In cis* mechanisms are those that involve the main cellular population that is targeted by ipilimumab: T cells that express CTLA-4 and therefore are restrained by a suppressive brake [6]. CTLA-4 blockade releases their brake and allows them to be activated, proliferate and carry out their effector functions. *In trans* mechanisms involve additional populations [7], mainly regulatory T cells and myeloid derived suppressive cells (MDSCs), and their suppressive potential can be diminished as a result of treatment [8]. To fully understand both types of mechanisms is crucial since it could lead to a better prediction of treatment outcome.

The use of non-cryogenically stored samples is becoming increasingly important to analyze key cellular populations [9–12]. To our knowledge, this is the first study focused on myeloid and lymphoid populations in which freshly isolated blood samples from ipilimumab treated patients were analyzed. This allowed us to precisely interrogate the effect of CTLA-4 blockade on different cell populations which are sensitive to freezing such as MDSCs, particularly those of polymorphonuclear origin [13].

The main objective of this study was to evaluate changes in the immune system of patients undergoing treatment with ipilimumab, with the prospect of elucidating the mechanisms involved in response to the treatment and their possible relations to clinical outcome. To do this we analyzed cellular populations and immune-related phenotypic markers from fresh peripheral blood samples taken in patients with advanced melanoma before and during ipilimumab treatment.

## RESULTS

### Treatment outcome and patient evaluation

Detailed information on the 43 patients included in this study can be found in Table 1. The follow-up time was between 45 and 227 weeks. The median overall survival (MOS) was 39 weeks. The objective response rate was 19%, with no patients obtaining a complete response, 8 (19%) patients achieving a partial response, while 9 (21%) patients were classified as having stable disease, 24 (56%) progressive disease and 2 patients (4%) were non evaluable. For analytic purposes, patients were divided into two groups: 17 (41%) patients with clinical benefit (includes responders and patients with stable disease) and no clinical benefit (23 patients with progressive disease). Patients with clinical benefit had an MOS of 80 weeks, significantly longer than the 23 week MOS in the no clinical benefit group (p < 0.0001) (Supplementary Figure 1).

**Table 1:** Patient Characteristics

Variable	n (%)
**Patients**	43
**Age**	
>65	23 (53)
<65	20 (47)
**Sex**	
Female	11 (26)
Male	32 (74)
**Primary**	
Cutaneous	26 (61)
Unknown	10 (23)
Mucosal	4 (9)
Uveal	3 (7)
**Stage**	
IV	43 (100)
M1a	9 (21)
M1b	6 (14)
M1c	28 (65)
**Braf status**	
Positive, V600E	10 (23)
Wild type	32 (75)
Unknown	1 (2)
**LDH**	
Normal	17 (40)
Elevated	22 (51)
Not evaluated	3 (7)
NA	1 (2)
**Line of treatment**	
First line	19 (45)
Second line	16 (37)
Third line	7 (16)
Fourth line	1 (2)
**CNS metastases**	11 (25)
Treated with SABR	9 (20)
Treated with HBRT	1 (2)
No treatment	1 (2)
**Corticosteroids**	5 (11)
Low dose	5 (11)
High dose	0

In total, 64% (28) of the patients received all four cycles, 20% (9) three cycles and 16% (7) 2 cycles. The most frequent reason for discontinuation was adverse events. Thus, 53% (23) of the patients experienced adverse events of any grade, the most frequent being diarrhea/colitis. Moreover, 26% (11) of the patients experienced adverse events of grade 3 or higher, 10 of them receiving treatment with high dose corticosteroids intravenously and 2 with anti-Tumor Necrosis Factor alfa (anti-TNF-α) therapy intravenously.

### Hematological measurements

**Figure 1:**
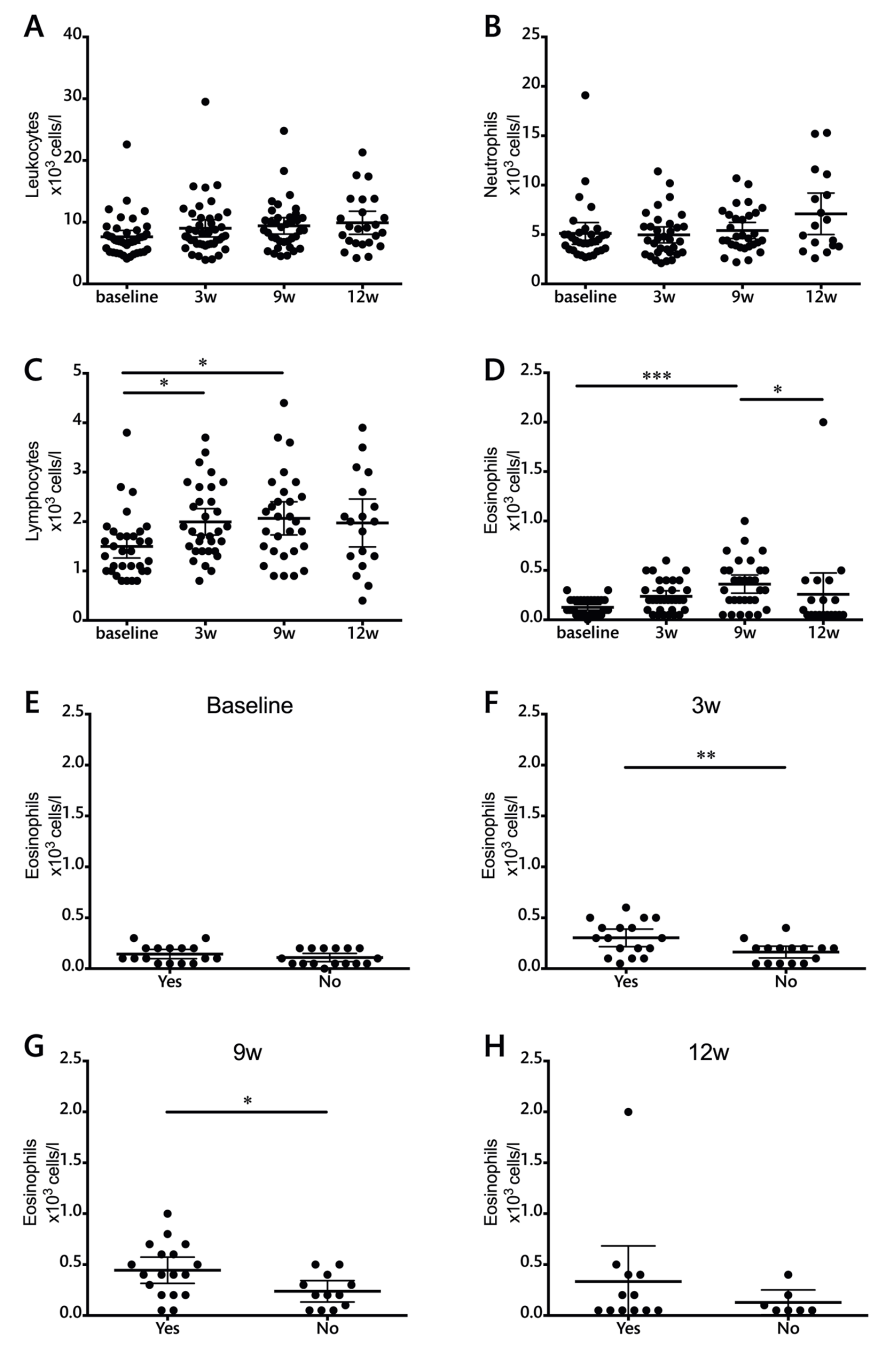
Total leukocyte and leukocyte subset counts during ipilimumab treatment (cells/l) **A.** leukocytes, **B.** neutrophils, **C.** lymphocytes, **D.** eosinophils. **E-H.** Evaluation of eosinophil counts in patients with or without adverse events at each individual time point. Each dot represents an individual patient; mean ±95% confidence interval (CI) are represented. *, P<0.05; **, P<0.001; ***, P<0.0001

The results of absolute cell counts in peripheral blood are shown in Figure 1. No changes were observed in the total leukocyte or neutrophil populations during ipilimumab therapy. Ipilimumab treatment, however, resulted in an increase in the absolute lymphocyte count. This increase was significant already at the earliest time point and throughout the treatment schedule. Eosinophil counts also increased during treatment although statistical significance was only observed at the latest time point. Three weeks after the end of the treatment, at week 12, eosinophil levels had returned to baseline levels. The absolute counts of eosinophils in peripheral blood at the 3- and 9-week time points were significantly higher in patients that presented immune related adverse events of any grade (Figure 1F and 1G).

None of these measurements showed significant differences between patients with progressive disease compared to those who obtained clinical benefit from the therapy (data not shown).

Serum lactate dehydrogenase (LDH) levels were also measured during treatment. Although overall values did not change during treatment (Supplementary Figure 2), a correlation between elevated LDH and lack of clinical benefit was observed at the 9-week time point (Supplementary Figure 2).

### Cryopreservation of samples alters frequencies of immune cell subsets

**Figure 2:**
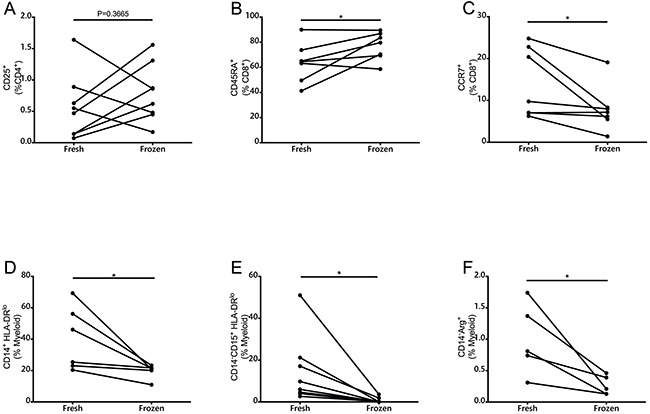
Comparison of immune parameters as measured by multicolor flow cytometry in fresh and frozen PBMCs **A.** CD25^+^ cells measured in the CD4+ population (n=8). **B.** CD45RA^+^ and **C.** CCR7^+^ cells in CD8^+^ T cells (n=6). **D.** MoMDSCs (n=6). **E**. PMN-MDSCs (n=6). **F.** CD14^−^Arg^+^ in myeloid cells (n=5). *, P<0.05

Depending on availability, six to eight patient samples were used for comparison between fresh and cryopreserved samples (Figure 2, Supplementary Figure 3). Significant differences were found in the frequencies of CD45RA^+^, CCR7^+^, MoMDSCs, PMN-MDSCs and Arg^+^ cells. CD25 expression was also compared between fresh and frozen samples and although no statistical significant differences were found, variation was substantial. CD4^+^ICOS^+^ cells and FoxP3 expression were also compared without finding differences between fresh and frozen samples in the frequency of this cellular population (data not shown). Due to the prominent differences observed when analyzing memory T cell frequencies, CD25 expression, and MDSCs, we decided to analyze exclusively fresh samples within two hours of blood extraction.

### CD4^+^ICOS^+^ T cells increase immediately after treatment

**Figure 3:**
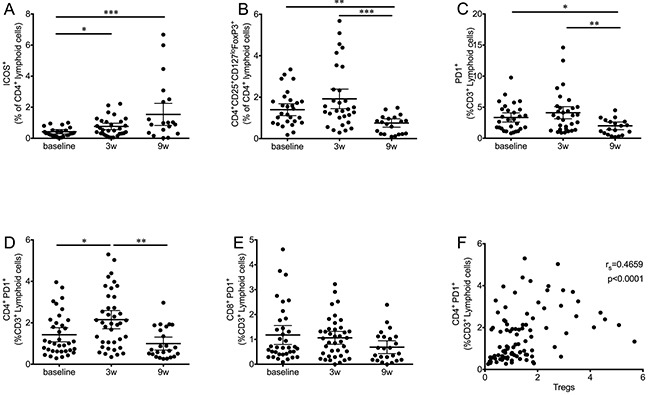
Kinetics of T cell subsets during ipilimumab treatment **A.** CD4^+^ICOS^+^ T cells significantly increase after the first ipilimumab dose and remained high at the end of treatment. **B.** Tregs (CD4^+^CD25^+^CD127^lo^FoxP3^+^) significantly decreased at the end of treatment after an initial increase. **C.** PD1^+^ T cells follow a similar pattern, an initial increase is followed by a significant decrease. **D.** The effect on PD1^+^ T cells is significant only in the CD4^+^ subset, while the decrease in CD8^+^ PD1^+^ T cells (E) is not significant. **F.** Correlation analysis of CD4^+^PD1^+^ with Tregs. Each dot represents an individual patient; mean ±95% CI are represented. *, P<0.05; **, P<0.001; ***, P<0.0001

Since the main target of CTLA-4 therapy is the activation of T cells, several activation markers were measured during the course of treatment. No significant changes were observed in the expression levels of CD28 or CD69 (data not shown). When the activation marker ICOS was measured in T cell populations, the first dose of ipilimumab was enough to significantly increase the frequency of CD4^+^ ICOS^+^ T cells (Figure 3A). This increase remained significant at the end of treatment, but showed no relation to survival (Supplementary Table 1).

### Overall decrease in circulating Tregs

In addition to T cell activation levels, we analyzed changes in the Treg population (CD4^+^CD25^+^CD127^lo^FoxP3^+^) during the course of therapy (Figure 3B). In this case, ipilimumab treatment resulted in a highly significant decrease in the frequency of this population at the nine-week time point. Early changes as a result of the first ipilimumab dose were not statistically significant, although an increase in data spread was observed.

### Modulation of PD1 expression in T cells

PD-1 is expressed upon T cell activation, in exhausted T cells and in Tregs [14–16]. When the expression levels of this marker were analyzed (Figure 3C), a similar pattern to that observed for frequencies of Tregs was observed: Overall, frequencies of CD3^+^ PD1^+^ cells were significantly decreased at the final time point, preceded by an increase three weeks after the first ipilimumab dose. Further analysis revealed that CD4^+^PD1^+^ T cells were responsible for the main changes observed in the total T cell population. Figure 3D shows that the first dose of ipilimumab results in a significant increase of CD4^+^PD1^+^ T cells, followed by a highly significant decrease at the final time point. The overall frequency of CD4^+^PD1^+^ T cells was decreased when compared to baseline levels, although this difference was found to be statistically significant only when matched analysis was performed with complete data sets. In contrast to the dynamics of CD4^+^PD1^+^ T cells, a strong trend towards a decrease in the CD8^+^PD1^+^ T cell population was observed (Figure 3E).

The populations of PD1^+^ T cells were analyzed for correlation with Treg frequencies. In this case, a moderate but highly significant correlation (r_s_=0.4659, P<0.0001) was found between Tregs and CD4^+^PD1^+^ (Figure 3F), but not CD8^+^PD1^+^ (not shown).

### Central memory and effector memory CD4 T cells are increased during treatment

**Figure 4:**
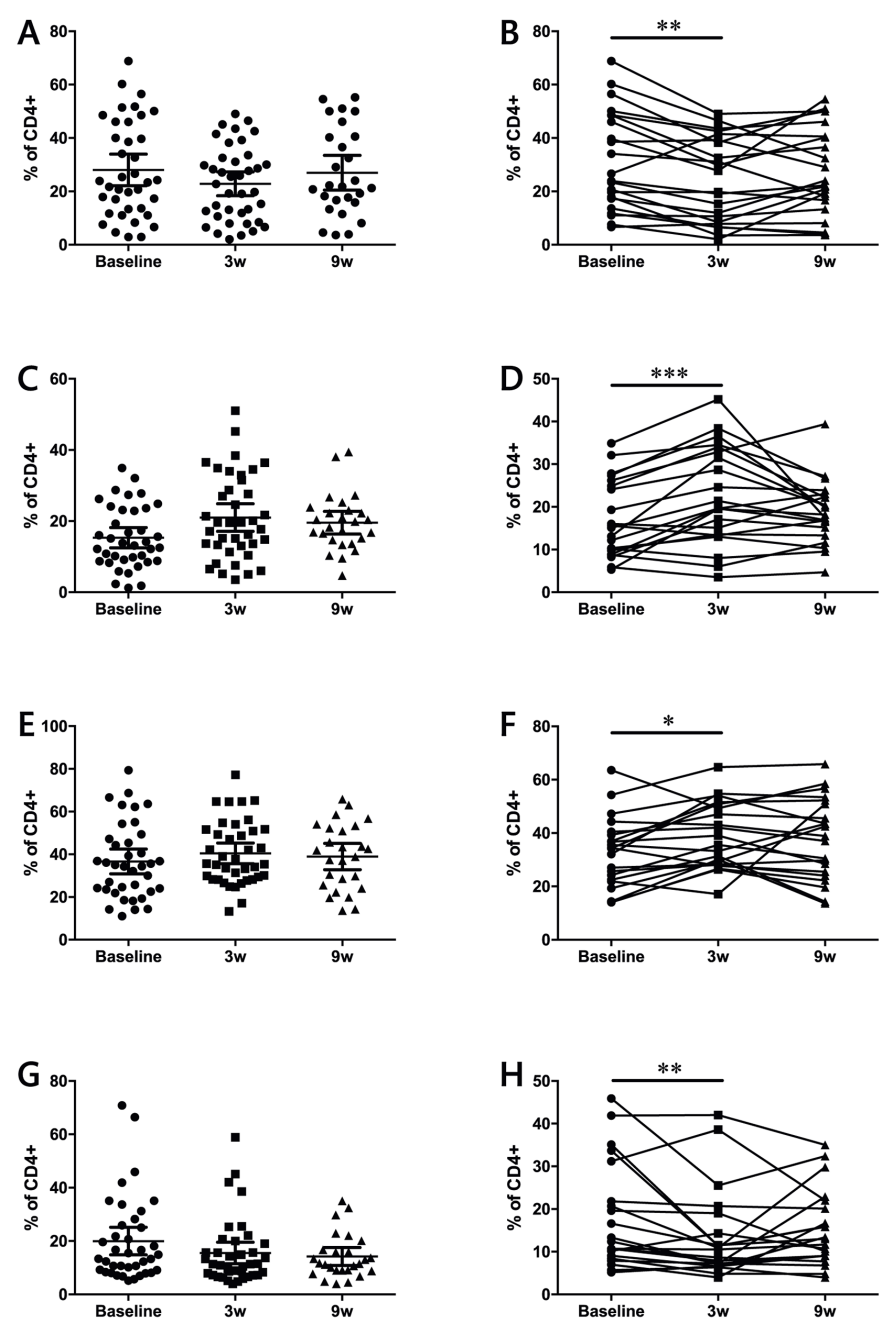
Effects of ipilimumab treatment on naïve (A-B), CM (C-D), EM (E-F) and EMRA (G-H) CD4+ T cell memory subsets (A-D) data from the full patient cohort; (E-H) when possible, matched analysis of complete sample sets (baseline, 3w and 9w) was performed. Each dot represents an individual patient; mean ±95% CI are represented *, P<0.05; **, P<0.001; ***, P<0.0001

CD45 RA and CCR7 are markers that can be used to subdivide CD4 and CD8 populations into naïve and memory subsets [17]. According to this classification, naïve T cells are CD45RA^+^CCR7^+^, central memory (CM) T cells lose CD45RA expression but still home to the lymph nodes (CD45RA^−^CCR7^+^), effector memory (EM) T cells are CD45RA^−^CCR7^−^, and terminally differentiated T cells (EMRA) are CD45RA^+^CCR7^−^. Analysis of these populations yielded different results for the CD4 and the CD8 subsets. In the case of CD4 (Figure 4), matched analysis of complete patient data sets revealed that naïve and EMRA cell frequencies decrease immediately after the first ipilimumab dose. This decrease is concomitant with an increase in the CM and EM populations. When the dynamics of memory subsets were analyzed in CD8 T cells, no overall changes were observed (Supplementary Figure 4).

### Ipilimumab treatment decreases suppressive potential in the myeloid compartment

**Figure 5:**
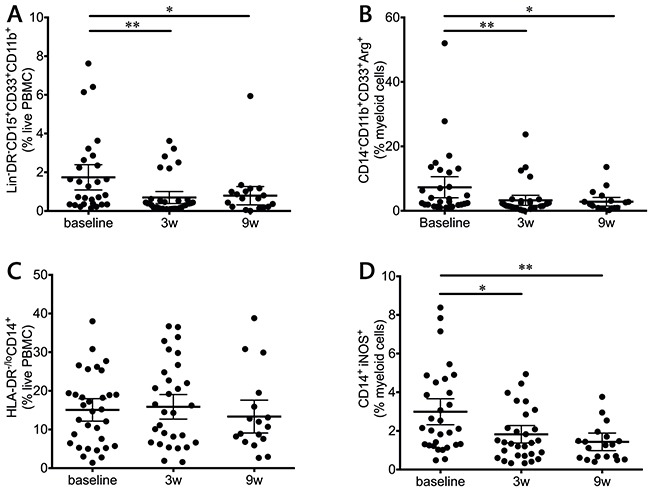
Myeloid populations in response to ipilimumab treatment PMN-MDSCs (Lin^−^CD14^−^CD11b^+^CD33^+^CD15^+^HLA-DR^lo/neg^) **(A)** and CD14^−^CD11b^+^CD33^+^Arg1^+^ **(B)** myeloid cells are decreased during treatment. **C.** Overall MoMDSCs (CD14^+^HLA-DR^lo/neg^) during treatment. **D.** iNOS+ monocytic cells are decreased during treatment.

MDSCs are highly suppressive in melanoma, and have been inversely correlated with MOS [18, 19]. Two MDSC populations were measured in this study (Figure 5): monocytic (MoMDSC, CD14^+^HLA-DR^lo/neg^) and polymorphonuclear (PMN-MDSC, Lin^−^CD14^−^CD11b^+^CD33^+^CD15^+^HLA-DR^lo/neg^). Ipilimumab significantly decreased the frequency of PMN-MDSC after the first dose (Figure 5A), and these levels remained low during the course of treatment. On the other hand, no treatment effects were observed on the MoMDSC population (Figure 5C).

MDSCs can suppress T cells via a broad array of mechanisms, one of which includes arginine depletion via the production of the arginine-degrading enzymes arginase-1 (Arg1) and inducible Nitric oxide synthase (iNOS) [20]. When intracellular levels of these enzymes were measured, CD14^−^CD11b^+^CD33^+^ cells showed a highly significant decrease in Arg1 (Figure 5B) and CD14^+^ cells presented a decrease in iNOS levels (Figure 5D). In both cases, the decrease was significant after the first infusion and remained significant at the end of the treatment.

### MoMDSCs are correlated with clinical benefit and survival

**Figure 6:**
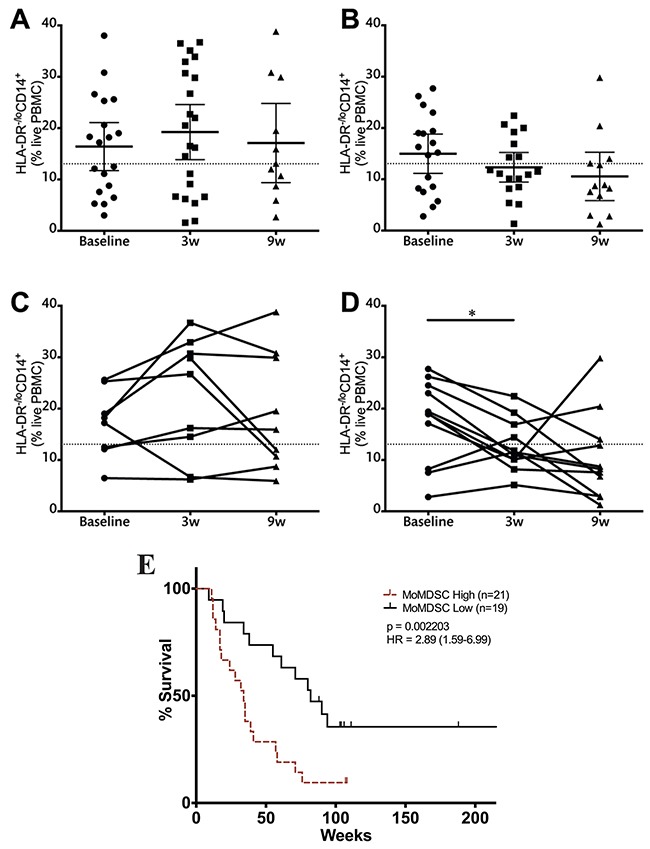
Monocytic MDSCs and clinical benefit Analysis of MoMDSCs at the three-week time point in patients with progressive disease **(A, C)** and clinical benefit **(B, D)** **(A-B).** data from the full patient cohort; **(C-D)** when possible, matched analysis of complete sample sets (baseline, 3w and 9w) was performed. Dashed line represents cutoff point (13.05%) that divides MoMDSC frequency into high and low as calculated using Cutoff Finder software. Each dot represents an individual patient; mean ±95% CI is represented. **E.** OS analysis according to MoMDSC frequencies at the three-week time point; censored patients are indicated by vertical lines. *, P<0.05; **, P<0.001; ***, P<0.0001.

MoMDSCs have previously been described as potential predictive biomarkers for treatment outcome [21–24]. When this cohort of patients was analyzed, no significant differences were found in baseline MoMDSC between patients with clinical benefit and those with progressive disease (Supplementary Table 1). In contrast to this observation, strong trends were observed at the three and nine-week time points, where patients with clinical benefit showed lower frequencies of this cellular population (Supplementary Figure 5). These results motivated further analysis, where the time course of the frequency of MoMDSCs was evaluated separately according to clinical response (Figure 6). The decreasing trend in MoMDSCs in patients with clinical benefit became more apparent and was statistically significant when pairwise analysis of fully matched sets was carried out (Figure 6A-6D): Patients with clinical benefit showed a significant reduction in the frequency of MoMDSCs in response to the first ipilimumab dose, at the three-week time point (Figure 6D). On the other hand, no significant changes were observed in patients with progressive disease. It is interesting to note that all patients with clinical benefit that presented MoMDSCs above the designated 13.05% cutoff point experienced a decrease in the frequencies of these cells in response to the first ipilimumab dose.

In view of this data, survival analysis was performed comparing patients with high and low MoMDSCs. The cutoff point, as determined using Cutoff Finder software, was 13.05%. No significant differences in survival were observed at baseline and nine week time points (data not shown). At the three-week time point, MoMDSCs showed to be inversely correlated with survival (Figure 6E).

### CD8 EM cells at the end of treatment can predict clinical outcome and correlate with survival

**Figure 7:**
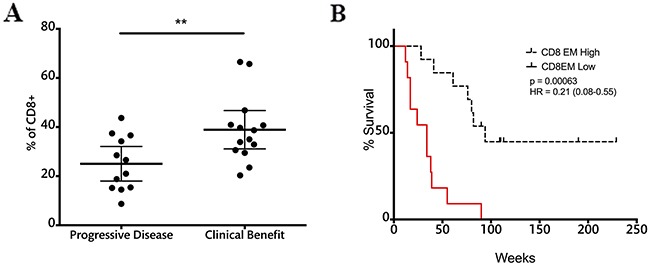
CD8EM cell frequencies at the end of treatment correlate with response and survival **A.** CD8EM frequencies in patients with progressive disease and clinical benefit; dashed line represents cutoff point (30.05%) that divides CD8EM frequency into high and low as calculated using Cutoff Finder software. Each dot represents an individual patient; mean ±95% CI is represented. *, P<0.05; **B.** OS analysis according to CD8EM frequencies at the nine-week time point; censored patients are indicated by vertical lines.

The frequencies of memory and effector T cells were also assessed in relation to clinical benefit after ipilimumab treatment. None of the subsets of CD4 cells were related to treatment outcome or survival (Supplementary Table 1). In the case of CD8 T cells, patients with clinical benefit had significantly higher effector memory cells at the end of treatment when compared to patients with progressive disease (Figure 7A).

Patients were divided into two groups according to the frequency of CD8 EM T cells using Cutoff Finder software. Survival analysis showed that patients with high CD8 EM cell frequencies above 30.05% at the nine-week time point had a MOS of 80 weeks versus 34 weeks in the low CD8 EM. Log rank analysis showed highly significant differences in survival between these two groups (Figure 7B).

## DISCUSSION

In this work, extensive immune monitoring was carried out in patients with advanced melanoma with the intent of shedding some light on the circulating cellular populations that are targeted by ipilimumab treatment, their evolution during treatment, and their possible correlations with treatment outcome and survival. The median overall survival (39 weeks) and the overall response rate (23%) are in agreement with what has been described in other patient cohorts. Combining patients with stable disease and patients with partial response in a “clinical benefit” group is supported by the fact that both groups showed no differences in MOS and additionally presented similar LDH levels. These levels were significantly lower in the clinical benefit group at the end of treatment when compared to the progressive disease patients.

Immune related adverse events are frequent in ipilimumab-treated patients; in the cohort included in this work more than half of the patients suffered any grade of adverse events and 25% of the patients suffered grade 3 or higher adverse events. They are mostly manageable and reversible; if recognized in time and treated with corticosteroids when indicated, adverse events do not cause long-term sequels [25, 26]. The results shown here suggest that a routine eosinophil count evaluation could be of great value in predicting the onset of side effects. Eosinophil levels were significantly higher in patients that developed adverse events, and the observed differences were significant already two weeks after the first ipilimumab dose, well before these toxicities appeared in the patients. Previous studies had shown high eosinophil infiltration in areas of rash [27], and the correlation between systemic eosinophil counts and adverse events has been previously suggested [28]. Our results could guide clinicians to monitor patients with high eosinophil counts more closely and be more active in making the decision to administer corticosteroids as soon as the adverse events set in.

To our knowledge, this is the first study in which lymphoid and myeloid populations from non cryopreserved patient PBMCs were used for immunophenotyping by flow cytometry during ipilimumab treatment. The advantages of this approach include the ability to study PMN populations that cannot resist freeze-thaw cycles [29], and avoiding potential frequency changes that appear after freezing in critical populations such as Tregs and MDSCs [9–13]. In particular, PMN-MDSCs are highly sensitive to freeze-thawing [13], and our finding of a dramatic decrease in this population already after the first dose of ipilimumab would not have been possible to make unless the analysis was carried out on fresh samples. Furthermore, the quality of the measurement of functional parameter Arg1 was similarly impaired, in agreement with previous studies by Kotsakis et al [12]. Our measurements show that MoMDSCs are also susceptible to freeze-thawing, something that should be taken into account in future studies of this population.

The inherent nature of CTLA-4 blockade implies that the T lymphocytes, being the main target cell population, should experience the most changes as a result of ipilimumab treatment. Monitoring T cells showed, along with an increase in the overall lymphocyte blood counts, an increase in activated CD4^+^ ICOS^+^ T cells accompanied by an increase in central and effector memory CD4 T cells, as previously reported [30]. Of special relevance was the fact that only one dose of ipilimumab was needed to induce a significant increase in these populations. These *in cis* effects on T cells were independent of treatment outcome and have been previously suggested as potential pharmacodynamic biomarkers [31]. The lack of changes in the overall CD8 subpopulations had been previously observed in frozen samples at later time points [30]. Our data confirms that ipilimumab may be acting preferentially on CD4 T cells, which are known to express higher levels of CTLA-4 [32].

Patients with advanced melanoma have been reported to have high frequencies of Tregs and MoMDSCs [33] that can be highly immunosuppressive [18] and impede the development of an effective immune response. In this study, ipilimumab treatment significantly reduced the suppressive pressure from these populations, by reducing both their frequency and their potential suppressive mechanisms. Tregs were decreased, but only at the end of treatment; Tregs have been considered an ideal target for CTLA-4 blockade therapy, since they constitutively express high levels of CTLA-4 [34]. One of the mechanisms that may be involved in this decrease is ipilimumab-mediated ADCC, as has been shown in mouse models [35] and in *in vitro* studies with human Tregs [36]. In addition to this decrease in Tregs, myeloid populations, which have also been described to be CTLA-4^+^ [37–39], were also affected by treatment. In this case the decrease in PMN-MDSCs and Arg1^+^ myeloid cells took place at an earlier stage of treatment, during the two weeks that followed the administration of the first ipilimumab dose. It is interesting to note that overall frequencies of MoMDSCs did not change during this study, although a significant decrease was observed in the iNOS production of monocytic cells. Again, this decrease takes place already after the first dose of treatment, making it possible that the overall decreases in MDSC frequency and suppressive potential may be an additional cause of the decrease in Tregs. Treg generation may be influenced by MDSCs as has been suggested by several studies [40–42], and Arg1 has been directly pointed out to be responsible for Treg development in a preclinical B cell lymphoma model [43].

Another cellular population that was affected by CTLA-4 blockade was the PD1^+^ T cell population. Surface PD1 expression is increased upon activation on T cells [44] rendering them susceptible to suppression via the PD1/PDL-1 pathway [45]. We show that at the end of treatment, the overall frequency of PD1^+^T cells is significantly decreased. In addition, this pathway is known to be crucial in the development of Tregs [46, 47], so the parallelism between the PD1^+^ T cell population and the Treg population was expected. When PD1 expression was analyzed in T cell population subsets, the differences between CD8 and CD4 T cells were prominent. As in the memory populations, the effect was statistically significant only in the CD4 populations. In this case, PD1^+^CD4^+^ T cells were significantly increased immediately after the start of treatment, which could indicate an increase in the activation status of the CD4 cells in response to the treatment. This increase might shed some light on the optimal time for combining anti-CTLA-4 with anti-PD-1 therapy, indicating that this combination could be more effective if PD-1 blocking antibodies are administered at the time of PD-1 increase.

As a summary of the pharmacodynamic effects that were observed in this cohort, an increase in activation of T cells is accompanied by a release of the brake that is set by MDSCs and Tregs upon the immune system of patients with advanced melanoma. These two effects may be necessary, but are not sufficient for patients to respond to treatment. The mechanisms by which they are induced should be further explored, in particular those related to the means by which ipilimumab interacts with MDSCs remain to be elucidated. The changes in these cellular populations should be confirmed in a validation cohort and compared with the fluctuations of an untreated melanoma patient cohort before being considered as pharmacodynamic biomarkers.

A lower MoMDSC frequency at baseline has recently been associated with treatment outcome in three independent studies [21–23]. In addition to this, MoMDSCs have previously been inversely correlated with overall survival in patients with advanced melanoma regardless of therapeutic approach [48]. This cellular population is correlated with disease severity: those patients with higher MoMDSC frequencies have a higher tumor burden [18] and therefore lower chances of responding to any type of therapy. In our patient cohort we did not, however, observe any correlation between baseline MoMDSCs and survival or treatment outcome. The correlation was found three weeks after the first ipilimumab infusion when patients that benefited clinically from treatment had lower MoMDSCs after the first infusion. Furthermore, when the patient cohort was stratified into MoMDSC high and MoMDSC low at the three-week time point, there was a 46 week difference in MOS. The correlations of MoMDSC with survival and clinical benefit at the three-week time point are in agreement with the observation that only patients with clinical benefit from ipilimumab treatment exhibited a significant reduction in the frequency of this cellular population. This suggests that a clinical response to ipilimumab treatment requires an effective remodeling of the suppressive compartment of the immune response.

Of similar importance is the relationship between CD8 effector memory T cells, clinical benefit and survival. In this case the significant differences were observed when patients received their last ipilimumab dose. It is logical to assume that the increased CD8 EM population may be responsible for tumor control and directly correlated with an increased survival. While CTLA-4 blockade had no effect on overall CD8 populations, the increase in CD8 EM cells in patients with clinical benefit was preceded by the decrease in MoMDSCs. It is possible that the systemic release of suppressive pressure on the immune system after this decrease of MoMDSCs allows CD8 EM T cells to increase in frequency and exert their effector function. In this case, the effect of ipilimumab on CD8 T cells would be *in trans*, and only becomes apparent in responders as a consequence of an increased CD4 T cell activation and a decrease in suppression exerted by myeloid populations.

Our results emphasize the importance of early patient monitoring, as it may open the opportunity for clinical decisions to be made within three weeks after treatment has been started: a hypothetical patient with high eosinophil counts and high frequencies of circulating MoMDSC will be more prone to develop adverse events and at the same time less prone to clinically benefit from ipilimumab. In this situation, clinicians may have the opportunity to modify the therapeutic approach even before disease progression is observed.

Although ipilimumab is mainly targeted to act directly on T cells, these findings show that myeloid cells are clearly affected by treatment, highlighting the necessity of comprehensive immune monitoring that spans a broad set of immune markers. Understanding the key cellular populations involved in a beneficial response to ipilimumab treatment is the first step towards dissecting the fine mechanisms by which checkpoint blockade releases the brake on the immune system. We have shown in this work that the use of fresh samples from ipilimumab treated patients allows for precise quantitation of several cell types that would not be possible if analyzed otherwise. Among these populations, MoMDSCs and CD8 EM T cells may be essential for an adequate response to treatment, leading to clinical benefit and an increased overall survival.

## PATIENTS AND METHODS

### Patients

43 patients aged 23 to 80 years with metastasized stage IV (defined according to American Joint Committee on Cancer, AJCC) malignant melanoma gave written informed consent to participate in blood sample collection in connection with ipilimumab treatment at the Department of Oncology, Karolinska University Hospital, Stockholm, Sweden. The first 6 patients were included in a multicenter randomized double blinded clinical trial, CA184-169 sponsored by BMS (Bristol-Myers Squibb), receiving ipilimumab intravenously at 3 mg/kg or 10mg/kg every three weeks for up to four doses. Patients were unblinded with respect to dose level delivered after the end of the clinical trial. The remaining 38 patients received ipilimumab according to clinical routine intravenously at 3 mg/kg every three weeks for up to four doses.

The Stockholm Regional Ethics Committee approved the protocol and all patients provided written informed consent in accordance with the Declaration of Helsinki.

All patients had AJCC stage IV malignant melanoma (cutaneous, uveal, mucosal or unknown primary) and the majority had metastatic class M1c, Eastern Cooperative Oncology Group (ECOG) performance status score of 0-1.

Patient samples were collected between July 2012 and May 2015. Data from 8 of the patients in this study was used in a previously published report [8].

### Assessments

According to clinical routine, blood samples including complete blood count, electrolytes, creatinine, liver status and thyroid status were assessed before each dose along with the performance status of the patients and any adverse events before each new dose was administered. Approximately one month after the last dose of ipilimumab the patients underwent the first computer tomography (CT) for tumor response evaluation. Radiological tumor response evaluation for the first 6 patients participating in the trial CA184-69 was made according to WHO criteria. Radiological response evaluation for the remaining 38 patients was made in general according to immune related Response Criteria, irRC, although not as part of a formalized research follow up protocol. Severity of adverse events was graded according to the National Cancer Institute Common Terminology Criteria for Adverse Events version 4.0 (CTCAE 4.0).

### Peripheral blood samples

Peripheral blood samples were obtained at three time points during treatment. The first sample (baseline) was drawn immediately before the first ipilimumab infusion; the second sample (3 weeks) was obtained immediately before the second infusion, the third one was obtained immediately before the fourth and last infusion (9 weeks). Counts of leukocytes, neutrophils, lymphocytes and eosinophils were measured as per Karolinska Hospital guidelines and are expressed as cells/l.

Peripheral blood mononuclear cells (PBMCs) were obtained from the blood samples by ficoll density gradient centrifugation within 1 hour of sample collection (Ficoll-Paque plus, GE Healthcare Life Sciences 17-1440-02). Purified PBMCs were used immediately for flow cytometry staining and analysis or cryopreserved in fetal calf serum with 10 % DMSO.

### Antibodies and flow cytometry

Fresh PBMCs were stained according to the manufacturer's recommendations, after proper titration in order to obtain an optimal signal to noise ratio. Antibody details are provided in Supplementary Table 2. Dead cells were excluded with the LIVE/DEAD^®^ Fixable Aqua Dead Cell Stain Kit (Thermo Fisher Scientific L34957). Cells were analyzed using an LSRII flow cytometer (BD Biosciences) and FlowJo v10.1 software (Treestar, Ashland, OR), using a non-stained control for each sample and fluorescence minus one controls for critical stainings (FoxP3 and arginase-1). The gating strategy used for each of the populations analyzed in this study is shown in Supplementary Figures 6-8. Quality control of the flow cytometer's performance and CV values were monitored on a day-to-day basis using CS&T beads (BD Biosciences).

### Statistical analysis

Data is shown as mean with 95% confidence interval for each time point. All data sets were checked for normality (Shapiro Wilk test) and variance homogeneity (Bartlett's test). Two-tailed unpaired T test with Welch's correction and ANOVA followed by Holm-Sidak's multiple comparison test were employed. If non-parametric analysis was considered necessary, the tests used were Kolmogorov-Smirnov test (two-tailed) or Kruskal Wallis ANOVA followed by Dunn's multiple comparison test. Spearman's non-parametric test was used for correlation analysis.

Overall Survival (OS) was defined as the interval between treatment start to the time of last follow up or death. Survival probabilities were determined by Kaplan Meier analysis and curve comparison was analyzed by log rank tests. Cutoff points for survival analysis were determined using Cutoff Finder software [49]. In these cases, statistical significance was calculated with Bonferroni correction according to the number of comparison tests determined by the software.

All statistical analyses were conducted using GraphPad Prism version 6.00 for Windows (GraphPad Software) and IBM SPSS Statistics v23 (IBM corporation). Differences were considered significant when p≤0.05.

## SUPPLEMENTARY MATERIALS FIGURES AND TABLES



## Abbreviations

ADCC: Antibody-dependent cellular cytotoxicity
ANOVA: Analysis of variance
Arg1: Arginase-1
CI: Confidence interval
CM: Central memory
CT: computer tomography
CTLA-4: Cytotoxic T lymphocyte-associated antigen 4
CV: Coefficient of variation
ECOG: Eastern cooperative oncology group
EM: Effector memory
EMRA: Terminally differentiated
iNOS: Inducible nitric oxide synthase
LDH: Lactate dehydrogenase
MDSCs: Myeloid derived suppressor cells
MoMDSCs: Monocytic MDSCs.
PBMCs: Peripheral blood mononuclear cells
PD1: Programmed cell death protein 1
PMN-MDSCs: Polymorphonuclear MDSCs
