# Supplementary material for: Ipilimumab treatment decreases monocytic MDSCs and increases CD8 effector memory T cells in long-term survivors with advanced melanoma
Source: Oncotarget. 2017 Feb 16;8(13):21539–53. doi: 10.18632/oncotarget.15368 (PMC5400604; doi:10.18632/oncotarget.15368)
Supplement: Supplementary file 1 [file oncotarget-08-21539-s001.pdf]

Ipilimumab treatment decreases monocytic MDSCs and increases CD8 effector memory T cells in long-term survivors with advanced melanoma

SUPPLEMENTARY FIGURES AND TABLES

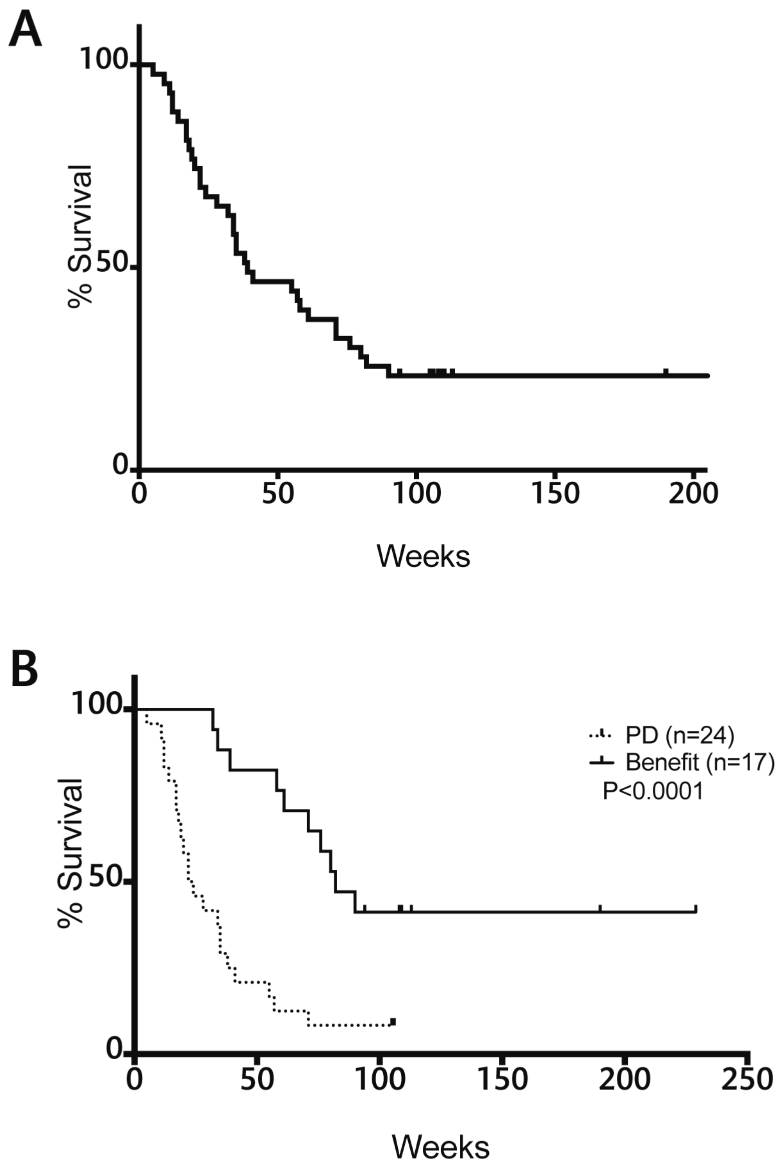

Supplementary Figure 1: Patient Survival. A. total patient survival. **B.** Comparison between the clinical benefit and the progressive disease groups

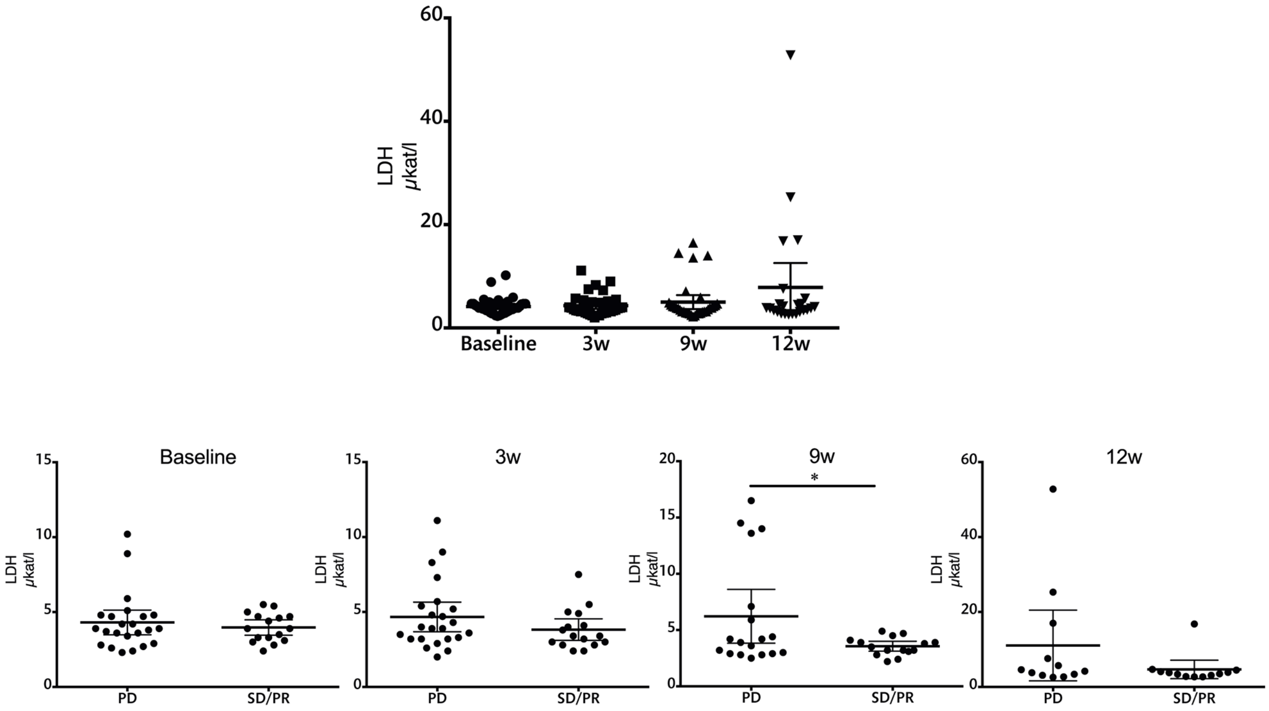

Supplementary Figure 2: Serum LDH activity (µkat/l) and correlation with clinical benefit during ipilimumab treatment. **A.** Baseline, **B.** three weeks after treatment start, **C.** nine weeks after treatment start and **D.** twelve weeks after treatment start. Each dot represents an individual patient; mean {plus minus}95% CI are represented *, P<0.05; **, P<0.001; ***, P<0.0001 *, P<0.05.

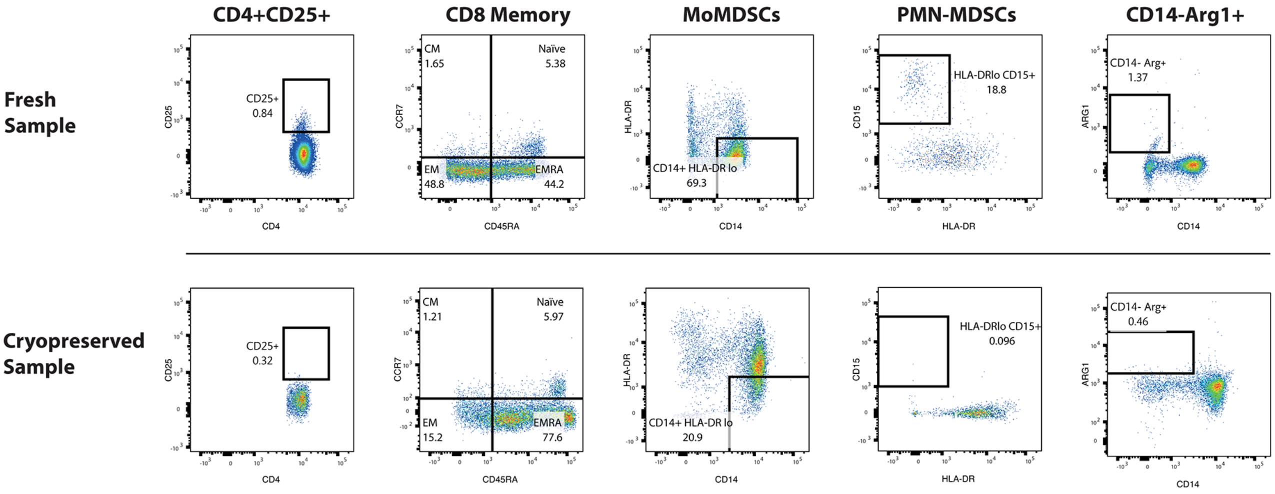

Supplementary Figure 3: Comparison between fresh and frozen samples.

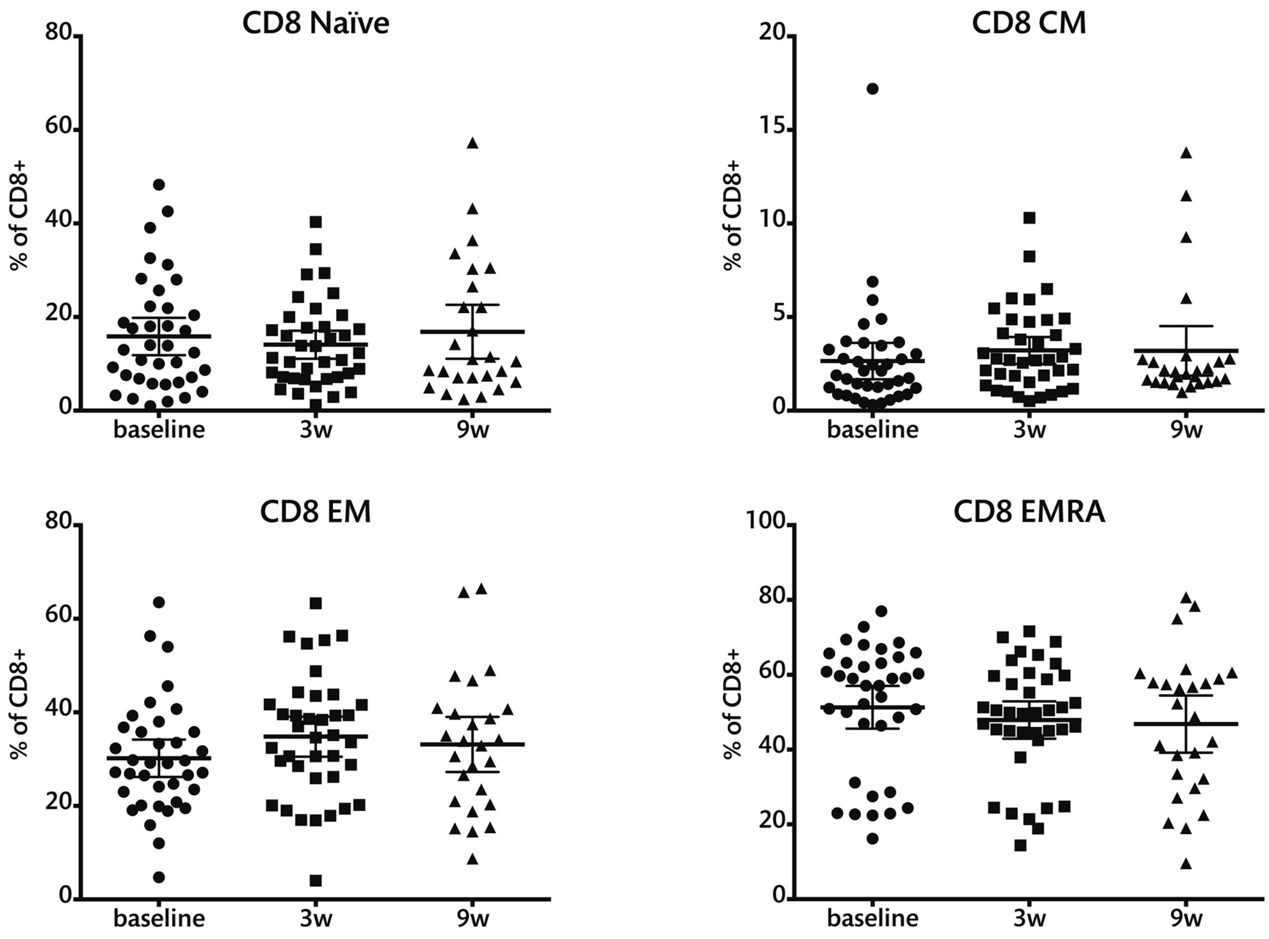

Supplementary Figure 4: Effects of ipilimumab treatment on naïve, CM, EM and EMRA CD8+ T cell memory subsets.

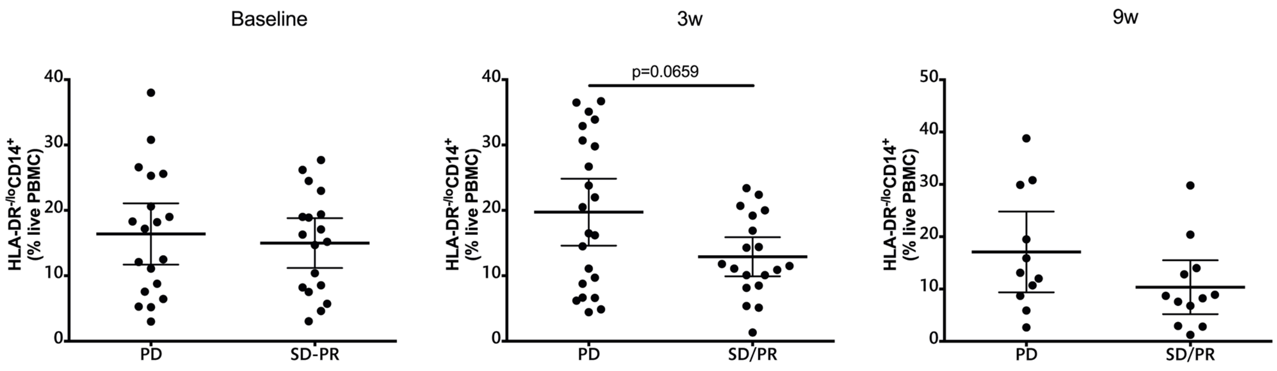

Supplementary Figure 5: Correlation between MoMDSC and clinical benefit at the different time points.

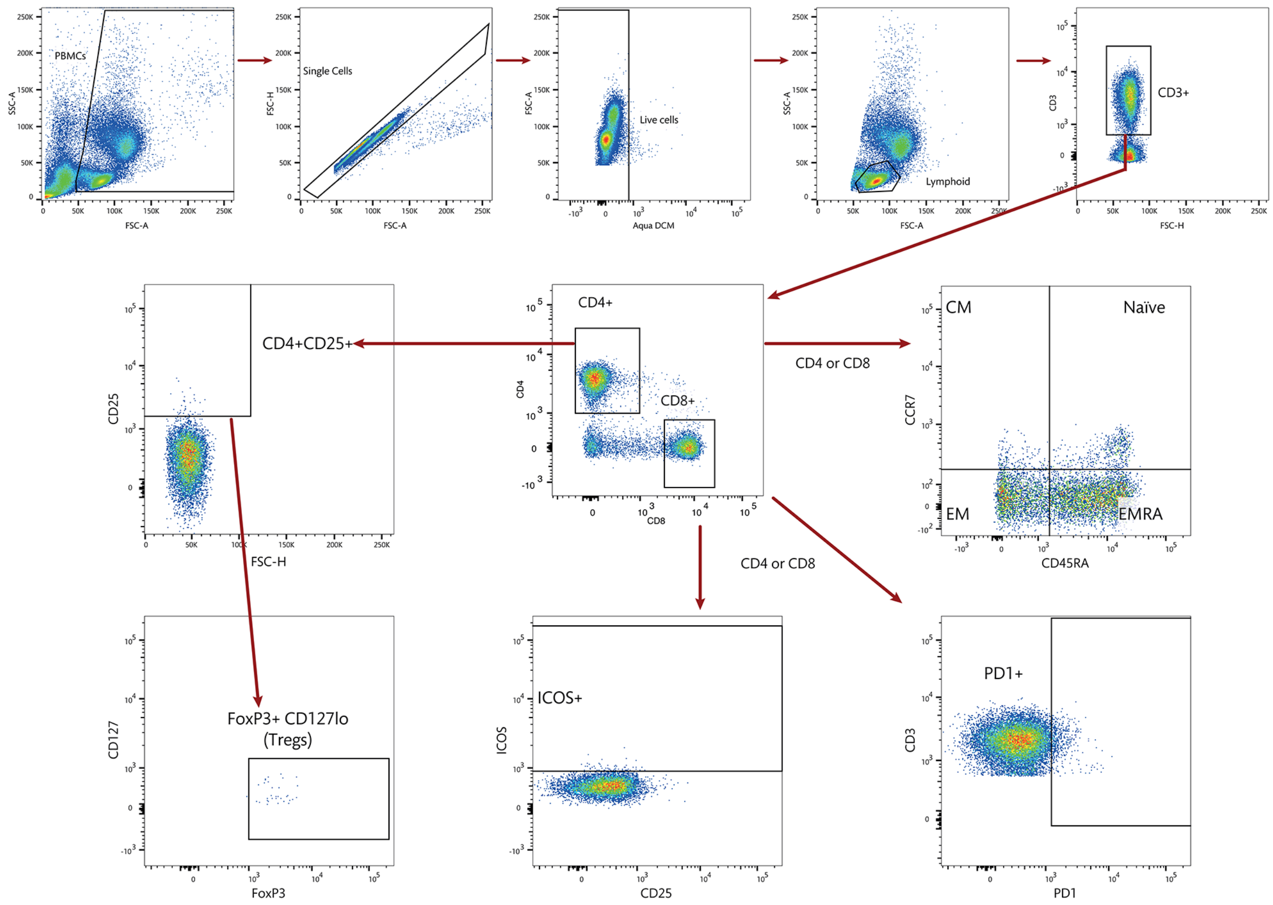

Supplementary Figure 6: Gating strategy for T cells.

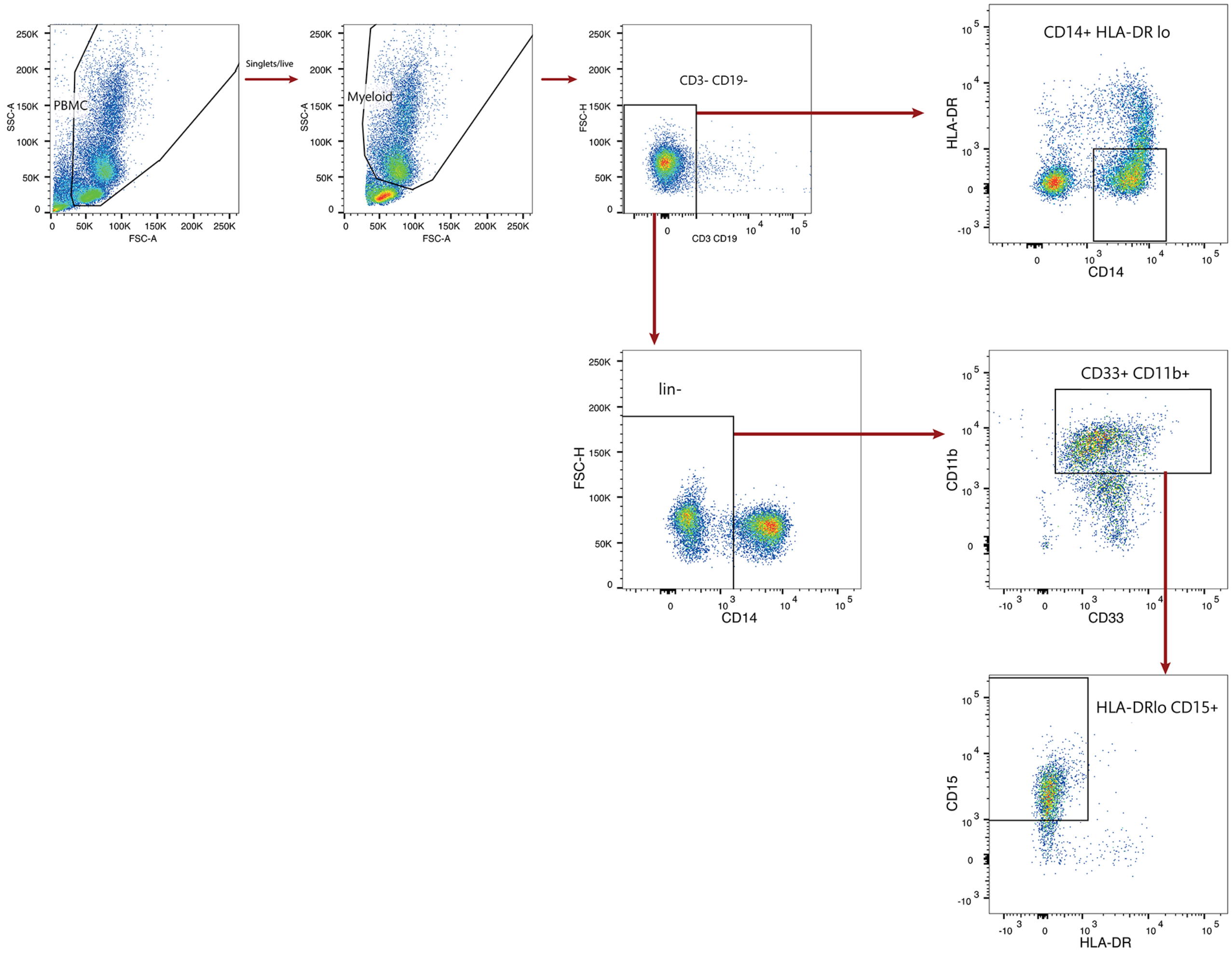

Supplementary Figure 7: Gating strategy for MDSCs.

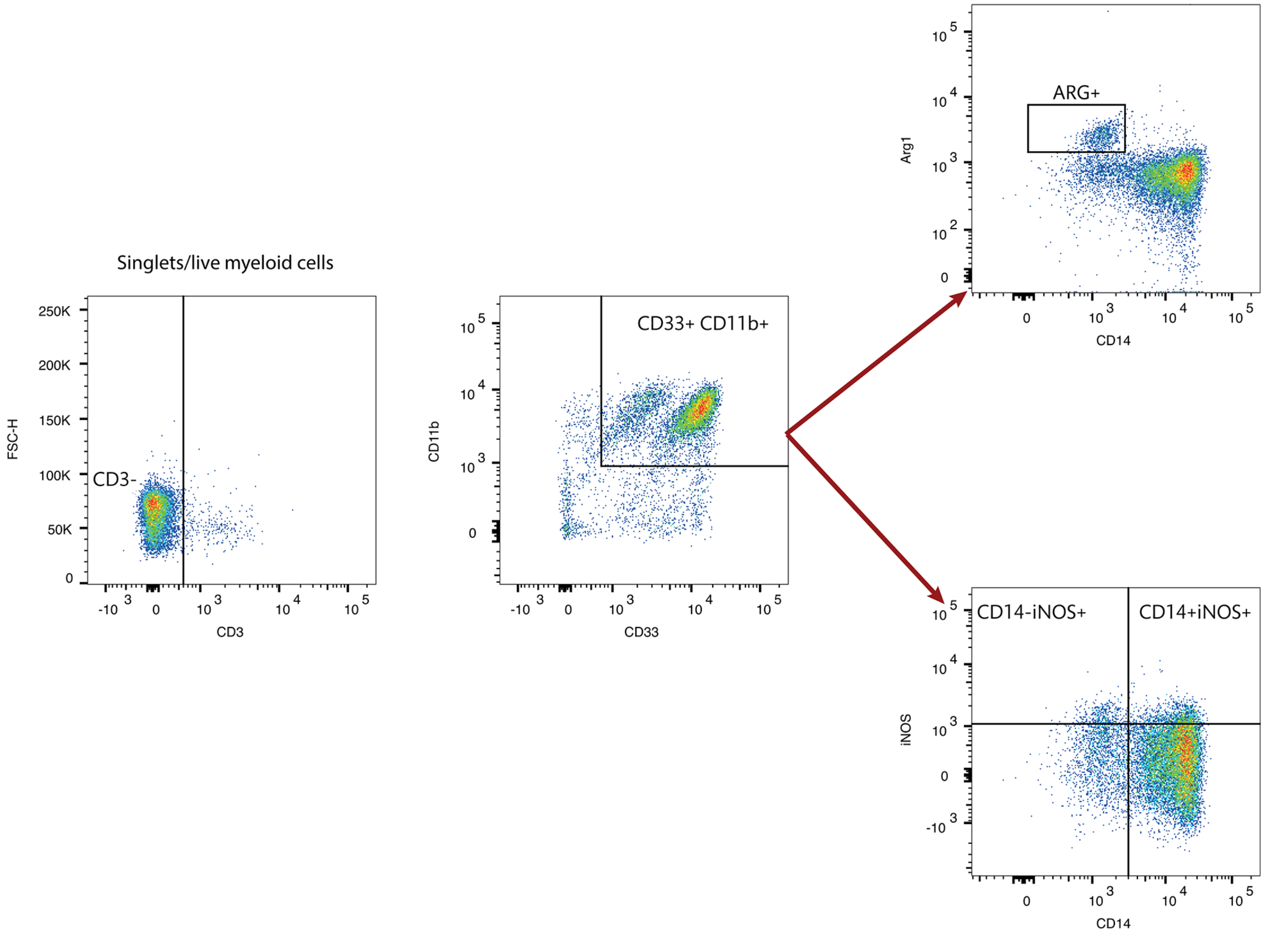

Supplementary Figure 8: Gating strategy for Arginase+ and iNOS+ myeloid cells.

Supplementary Table 1: Summary of analyzed cellular populations and correlation with survival

Population	Time point	Cut-off	p	HR
PMN MDSCs	Baseline	2.13	0.16	1.74 (0.79-3.81)
	3w	0.34	0.18	1.62 (0.8-3.3)
	9w	0.395	0.49	1.38 (0.55-3.51)
Arg	Baseline	3.69	0.11	1.79 (0.87-3.69)
	3w	3.02	0.11	0.48 (0.21-1.19)
	9w	1.52	0.79	1.13 (0.45-2.85)
MoMDSC	Baseline	18.6	0.29	0.67 (0.31-1.42)
	**3w**	**13.05**	**0.0022**	**2.89 (1.59-6.99)**
	9w	9.8	0.16	1.89 (0.76-4.69)
CD14+ iNOS	Baseline	1.965	0.25	1.55 (0.74-3.26)
	3w	1.38	0.06	2.03 (0.96-4.3)
	9w	1.285	0.28	1.64 (0.67-4.01)
Treg	Baseline	0.935	0.28	0.67 (0.32-1.4)
	3w	0.94	0.16	1.9 (0.77-4.68)
	9w	0.74	0.46	0.71 (0.29-1.77)
ICOS	Baseline	0.3	0.32	0.7 (0.34-1.44)
	3w	0.785	0.082	1.94 (0.91-4.12)
	9w	0.505	0.21	2.03 (0.66-6.26)
CD3 PD1	Baseline	2.05	0.19	1.62 (0.78-3.37)
	3w	3.79	0.19	1.64 (0.78-3.46)
	9w	1.39	0.27	1.72 (0.65-4.53)
CD4 PD1	Baseline	1.93	0.24	1.6 (0.73-3.53)
	3w	1.98	0.49	1.3 (0.62-2.72)
	9w	0.615	0.30	1.63 (0.64-4.16)
CD8 PD1	Baseline	1.35	0.46	0.74 (0.33-1.66)
	3w	1.04	0.25	1.53 (0.73-3.21)
	9w	0.355	0.19	1.9 (0.72-4.98)
CD4 Naive	Baseline	23.65	0.11	1.81 (0.86-3.83)
	3w	22.8	0.026[a]	2.36 (1.09-5.12)
	9w	23.1	0.046[a]	2.56(0.98-6.64)
CD4 CM	Baseline	13.45	0.38	1.38 (0.67-2.83)
	3w	13.15	0.11	2.05 (0.83-5.05)
	9w	16.85	0.13	2 (0.79-5.04)
CD4 EM	Baseline	44.85	0.37	1.43 (0.66-3.11)
	3w	42.6	0.11	0.53 (0.24-1.17)
	9w	40.4	0.34	0.65 (0.27-1.59)
CD4 EMRA	Baseline	14.15	0.22	0.63 (0.3-1.32)
	3w	11.4	0.25	0.64 (0.3-1.37)
	9w	11.6	0.035a	0.39 (0.16-0.96)
CD8 Naive	Baseline	15.55	0.23	0.64 (0.31-1.33)
	3w	8.965	0.42	0.74 (0.35-1.55)
	9w	8.425	0.41	1.47 (0.58-3.7)
CD8 CM	Baseline	1.89	0.35	1.42 (0.68-2.97)
	3w	2.145	0.055	2.22 (0.96-5.1)
	9w	1.895	0.37	1.51 (0.61-3.7)
CD8 EM	Baseline	34.65	0.26	0.63 (0.28-1.42)
	3w	28.65	0.082	0.51 (0.23-1.1)
	**9w**	**30.05**	**0.00063**	**0.21 (0.08-0.55)**
CD8 EMRA	Baseline	60.55	0.57	1.24 (0.59-2.62)
	3w	46.5	0.15	1.75(0.81-3.78)
	9w	40.15	0.42	1.46 (0.58-3.66)

Statistical significance after Bonferroni corrections is noted in bold.
[a]Not statistically significant after Bonferroni correction.

Supplementary Table 2: Antibodies used in flow cytometry.

Marker	Conjugate	Clone
Arg[1]	PO*	6G3
CCR7[2]	PE	150503
CD3[2]	FITC	UCHT1
CD3[3]	PerCP-Cy5.5	SK7
CD3[3]	PE-Cy7	UCHT1
CD4[4]	PE-TR	S3.5
CD8[4]	PE-Alf610	3B5
CD8[3]	APC-Cy7	SK1
CD11b[2]	APC-Cy7	ICRF44
CD14[3]	PB	M5E2
CD14[3]	PerCP-Cy5.5	M5E2
CD15[2]	PE-Cy7	HI98
CD19[2]	PerCP-Cy5.5	HIB19
CD25[3]	PB	BC96
CD28[3]	PerCP-Cy5.5	CD28.2
CD33[3]	APC	WM53
CD45RA[3]	APC	HI100
CD69[3]	APC-Cy7	FN50
CD127[3]	Alf647	A019D5
FoxP3[5]	FITC	PCH101
HLA-DR[3]	PE	L243
PD1[3]	PB	EH12.2H7

* conjugated with Pacific Orange Labeling Kit (Invitrogen, Carlsbad, CA)
[1]Hycult Biotech (Plymouth Meeting, PA), [2]BD Biosciences (San Jose, CA), [3]BioLegend (San Diego, CA), [4]Invitrogen (Carlsbad, CA), [5]eBioscience (San Diego, CA).
